# Progress and challenges to control malaria in a remote area of Chittagong hill tracts, Bangladesh

**DOI:** 10.1186/1475-2875-9-156

**Published:** 2010-06-10

**Authors:** Ubydul Haque, Masahiro Hashizume, Toshihiko Sunahara, Shahed Hossain, Syed Masud Ahmed, Rashidul Haque, Taro Yamamoto, Gregory E Glass

**Affiliations:** 1 International Center for Diarrhoeal Disease Research Bangladesh, 68 Shaheed Tajuddin Ahmed Sharani, Mohakhali, Dhaka 1212, Bangladesh; 2 Department of International Health, Institute of Tropical Medicine (NEKKEN) and the Global Center of Excellence Programme, Nagasaki University, Japan; 3 BRAC Research and Evaluation Division, BRAC Centre, 75 Mohakhali, Dhaka-1212 Bangladesh; 4 Department of Molecular Microbiology and Immunology, John Hopkins Bloomberg School of Public Health, Baltimore, MD 21205, USA

## Abstract

**Background:**

Malaria is endemic in 13 eastern districts where the overall infection prevalence is 3.97%. In 2006, Bangladesh received US$ 36.9 million from the Global Fund to Fight AIDS, Tuberculosis and Malaria (GFATM) to support the national malaria control programme of Bangladesh.

**Objectives:**

The objective of this study was to i) clarify factors associated with treatment seeking behaviours of malaria ii) distribution of LLIN, and iii) re-treatment of ITN in remote area of a CHT district of Bangladesh two years after implementation of national control programme.

**Methods:**

All households of Rajasthali sub-district of Rangamati district (households about 5,322, population about 24,097), all BRAC health workers (n = 15), health facilities and drug vendors' locations were mapped. Distances from households to health facilities, BRAC health workers and drug vendors were calculated. Logistic regression analysis was performed to assess the associations between the choice of the treatment and the distance to various treatment sources, education, occupation and ethnicity. SaTScan was used to detect clustering of treatment-seeking approaches.

**Findings:**

LLIN distribution and the re-treatment of ITN exceeded target goals. The most common treatment facility for malaria-associated fever was malaria control programme led by BRAC and government (66.6%) followed by the drug vendor (48.8%).

**Conclusion:**

Closeness to health facilities run by the malaria control programme and drug vendors were significantly associated with the choice of treatment. A high proportion of people preferred drug vendors without having a proper diagnosis. Drug vendors are highly patronized and thus there is a need to improve their services for public health good. Otherwise it may cause incomplete treatment, misuse of anti-malarial drugs that will contribute to the risk of drug resistance and jeopardize the present malaria control efforts in Bangladesh.

## Background

Malaria is estimated to be responsible globally for a million deaths every year, and even though 90% of mortality occurs in Africa, it remains a major health threat in South-Asian countries, including Bangladesh. There, it is endemic in 13 eastern districts where the overall infection prevalence is 3.97%. The infections are predominantly *Plasmodium falciparum *(90.12%) and *Plasmodium vivax *(5.3%), with 4.5% mixed infections. The overall prevalence in the Chittagong hill tracts (three south-eastern districts) is 11.7%, reaching 36% in a single sub-district, Rajasthali [1].

The malaria control programme in Bangladesh faces formidable challenges, including access to quality healthcare services, inadequately trained personnel, difficulty in travel, a lack of resources and education for the population at risk, and life styles depending on subsistence activities. Health facilities to manage severe malaria also are limited, surveillance is inadequate, and interventions are insufficient [2].

Many studies have described socioeconomic, demographic and environmental risk factors as part of malaria-related knowledge, attitude, and practices studies [3-9] and while bed nets are usually considered a protective measure, they may be used insufficiently [10] or reported as ineffective [11-15]. However, recent studies indicate that insecticide-treated nets (ITN) reduced 48-50% of malaria episodes [16] and are considered one of the most cost-effective health interventions against malaria [11,12].

Baseline information for the implementation of ITN has shown that people were aware of malaria infection, transmission, anti-malarial drugs and malaria control [13]. WHO has recommended full coverage of all people at risk in areas targeted for malaria prevention with long-lasting insecticide-treated nets (LLINs). Especially for endemic areas, LLINs should be delivered to all people and should initially focus on priority target areas [14].

Little is known about treatment-seeking behaviour among indigenous people infected with malaria in Bangladesh. In a baseline survey, treatment-seeking at hospitals was rare, self-treatment was common and people commonly took drugs without consulting a qualified doctor. In five south-eastern districts, 32.3% people preferred to get treatment from drug vendors [3]. The choice of treatment source was related to distance from hospital, disease type, patient's gender and parent's education level. People also preferred to receive malaria treatment from the nearest health workers [17]. These results were similar to previous studies [18] indicative of suboptimal treatment regimes.

In 2006, Bangladesh received US $ 36.9 million from the Global Fund to Fight AIDS, Tuberculosis and Malaria (GFATM) to support a national malaria control programme that would integrate rapid diagnosis tests (RDTs), new drug regimens (artemisinin-based combination therapy (ACT)), expanded distribution of LLIN, enhanced surveillance, vector surveillance and better documentation of activities. Bangladesh adopted artemether-lumefantrine (AL)(Coartem^®^) as a first-line treatment of *P. falciparum *malaria and has sought to provide early diagnosis and prompt treatment to 80% of malaria patients. Other intervention objectives included effective malaria prevention to 80% of the population at risk and to strengthen the malaria epidemiological surveillance system. It was estimated that 40% of the households in high risk areas had nets, 10% of which were insecticide treated. Through this grant, it was expected that 80% of households (1.7 million) would be covered with LLINs. Nets currently present in households would be treated and re-treated twice a year with insecticide [19].

BRAC and the Ministry of Health implemented the national malaria control programme under GFATM and BRAC would be responsible for supplying LLIN to 80% household, as well as deploying health workers in every union to provide RDT and AL at the grass root level. This study was undertaken in Rajasthali, where the highest prevalence of infection had been detected to clarify factors associated with treatment seeking behaviours of malaria, distribution of LLIN, and re-treatment of ITN in remote area of a CHT district of Bangladesh two years after implementation of national control programme.

## Methods

### Study area and population

The study was conducted in Rajasthali sub-district of Rangamati district situated in the south-eastern part of Bangladesh. The area of this sub-district is 145.04 km^2 ^[19]. The area is hilly and remote covered with forests and streams and estimated to have highest prevalence of malaria (36%) [1]. The population of Rajasthali is 24,097 living in 5,322 households. Our target was to cover all households in the sub-district.

### Survey instrument

A questionnaire was developed with household id, name, detailed address, treatment-seeking behaviour, preferred hospital, demographic structure, education, occupation and information on possession and type of bed net use. Geographic location recorded with GPS (global positioning system) receivers. GPS also was used to record the position of BRAC health volunteers' households, all hospitals, health clinics, NGO hospitals and drug vendors in Rajasthali and in adjacent sub-districts outside Rajasthali. The study was conducted between January to April, 2010. Please see the appendix for details (Additional file 1).

### Data management and cleaning

Data were entered in MS Excel 2007 checked for errors or inconsistencies and analysed. The locations (longitude and latitude) of all households were recorded using eTrex Venture single handheld GPS receivers. Administrative boundary data were obtained from the Local Government and Engineering Department (LGED) of the Government of Bangladesh. GPS records were imported in Arc GIS 9.3 software and checked on the polygon boundary map. All errors were checked at field level. Distances between points of interest (distance from households to every health facility) were calculated using planar straight-line distance [20].

### Statistical analysis

Data was analysed with STATA 10. After characterizations of the frequency distributions, logistic regression analysis was performed to assess the associations between treatment-seeking behaviour and the distance to the place for the treatment, educational level, occupation and ethnicity. All variables were incorporated in multivariate regression model. Odds ratios (ORs) and corresponding 95% confidence intervals (CIs) were estimated in bivariate and multivariate models.

### Spatial analysis

SaTScan (v. 07) was used to detect spatial clusters for treatment-seeking approach (settings: spatial analysis; Bernoulli probability model; Cartesian coordinates; no geographical overlap). Clusters were determined by calculating the maximum likelihood ratio and dividing the number of observed cases by the number of expected cases in each cluster. Simulated p-values were obtained using Monte Carlo methods with 9,999 replications.

### Ethical considerations

This study was reviewed and approved by both the research review committee and ethical review committee of ICDDR, B.

## Results

The population of Rajasthali was 24,097 with a slight preponderance of males (50.3%). There were six different ethnic communities in Rajasthali - Marma, Tripura, Tonchonga, Khiang, Chakma and Bengali. Marma was the predominant and Bengali was the second largest ethnic group. Almost half of the heads of household (46.90%) were illiterate, and only 5.70% of household heads had completed more than 10 classes of education. The majority (50.50%) of household heads were farmers (Table 1).

**Table 1:** Baseline characteristics of the study population

Characteristics	N	%
**Population**		
Male	12131	50.30
Female	11966	49.70
**Ethnicity**		
Marma	2369	44.50
Tripura	423	7.90
Tonchonga	855	16.10
Khiang	211	4
Chakma	35	0.70
Bengali	1429	26.90
**Having bednet**		
Yes	5236	98.38
No	86	1.62
**Number of bed nets**		
< 2	1254	23.60
≥ 2	4068	76.40
**Source of bed nets**		
BRAC	4795	90.09
Own initiative	2964	55.69
Other	946	17.80
**All family members sleep under bed nets**		
Yes	4323	81.22
No	999	18.88
**Bed net treatment**		
Yes	2181	40.98
No	3141	59.02
**Education (years)**		
0	2498	46.90
1-5	1167	21.90
6-10	1355	25.50
> 10	302	5.70
**Occupation**		
Farmer	2687	50.50
Day labor	918	17.20
Service	546	10.30
Business	141	2.60
Others	1030	19.40
**Treatment seeking behaviour**		
Control program	3552	66.60
Drug vendor	2600	48.80
Other	88	1.70
**Minimum distance from health facilities to households Health facilities by control programme**		
< 2 km	4149	77.95
≥ 2 km	1173	22.05
**Drug vendor**		
< 2 km	2858	53.70
≥ 2 km	2464	46.30

LLIN were provided to 90.09% of households by BRAC and 76.40% households had more than two bed nets. BRAC had treated bed nets in 40.98% of households in the six months prior to survey. It was found that 81.22% of people preferred to sleep under a bed net.

There were diverse treatment-seeking behaviours though nearly two thirds of people (66.60%) preferred to consult with trained health workers or doctors in a government hospital (control programme). A large minority of people (48.80%) also reported they received treatment from drug vendors. A small fraction (1.70%) preferred other choices (taking no measures, home-made medicine, kabiraj (who provide traditional herbal treatment in rural areas in Bangladesh), homeopathy and village doctors.

**Figure 1:**
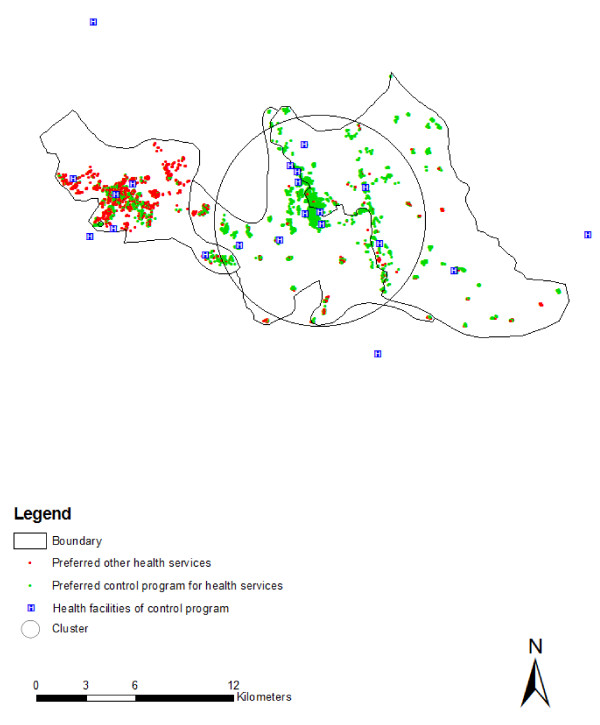
**Distribution of household clusters prefer health services from malaria control Program**.

**Figure 2:**
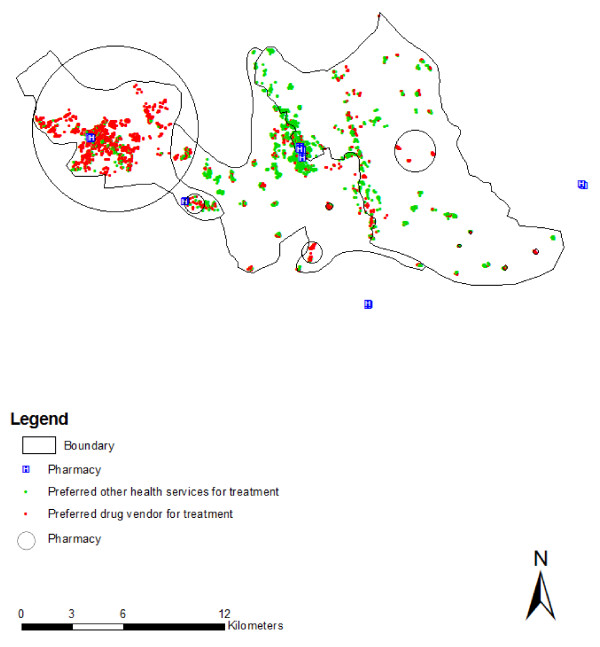
**Distribution of household clusters prefer health services from drug vendors**.

Access to care, as characterized by distance appeared to play a significant role in treatment preference. A large proportion of households lived within 2 km of health facilities supported by national malaria control programme (77.95%), as well as drug stores (53.70%) (Table 1). People who lived within 2 km of health facilities led by the malaria control programme preferred it compared with people who lived more than 2 km away from health facilities. As the distance increased (≥ 2 km), people were less likely to prefer the control programme's health facility (OR = 0.48; 95% CI: 0.40-0.08), but, instead, preferred drug vendors (OR = 2.02; 95% CI: 1.71-2.39). There were also ethnic differences in the utilization of health care facilities, study result confirm that people from Tonchonga ethnic community prefer most the malaria control program for treatment (OR = 7.07; 95% CI: 5.57-8.98) while people of Tripura ethnic community (OR = 3.12; 95% CI: 2.41-4.05), and Khiang ethnic community (OR = 2.46; 95% CI: 1.78-3.41) prefer the malaria control programme for treatment. Compared with other communities, Bengali people were more likely to seek treatment from drug vendors (Table 2). These heterogeneities were also associated with spatial clustering in health seeking approaches. There were two clusters where people preferred to use the malaria control programme. One was most likely cluster and another one was secondary cluster (Table 3; Figure 1). The most likely cluster was in the central-western part of the study area (RR = 2.24; P = 0.001). There were also several isolated villages in eastern part where people did not prefer treatment provide by malaria control programme. There was also a cluster favouring using drug vendors (RR = 2.97;P = 0.001) in the western part of Rajasthali. Eight secondary clusters were observed in the study area where people preferred drug vendors for treatment. Among them six were statistically significant (Table 3). All secondary clusters were in the southern and eastern part of Rajasthali (Figure 2). People who were involved in the service and business industries seemed to have a better treatment-seeking approach compared with people involved in agriculture.

**Table 2:** Treatment seeking behaviour in Rajasthali

	Bivariate				Multivariate			
	**Control programme**		**Drug vendor**		**Control programme**		**Drug vendor**	
**Variables**	**OR**	**95% CI**	**OR**	**95% CI**	**OR**	**95% CI**	**OR**	**95% CI**

**Control program**								
< 2 km.	1		1		1		1	
≥ 2 km.	0.60	0.53 - 0.69	1.70	1.50 - 1.95	0.48	0.40-0.58	2.02	1.71 - 2.39
**Drug vendor**								
< 2 km.	1		1		1		1	
≥ 2 km.	1.05	0.94 - 1.18	1.04	0.93 - 1.20	1.11	0.93-1.33	1.09	0.92 - 1.28
**Education (years)**								
0	1		1		1		1	
1-5	1.09	0.94 - 1.26	1.05	0.91 - 1.20	1.06	0.91 -1.25	1.03	0.88 - 1.19
6-10	1	0.87 - 1.15	1.08	0.95 - 1.20	1.06	0.90 -1.24	1.03	0.88 - 1.20
> 10	1.49	1.14 - 1.96	0.69	0.54 - 0.88	1.24	0.89 -1.74	0.84	0.62 - 1.14
**Tribe**								
Marma	1		1		1		1	
Tripura	3.01	2.36 - 3.85	0.46	0.38 - 0.57	3.12	2.41- 4.05	0.39	0.31 - 0.50
Tonchonga	7.17	5.67 - 9.06	0.35	0.29 - 0.41	7.07	5.57- 8.98	0.34	0.28 - 0.40
Khiang	2.13	1.56 - 2.90	0.27	0.19 - 0.37	2.46	1.78 - 3.41	0.21	0.15 - 0.29
Chakma	2.79	1.26 - 6.17	0.12	0.05 - 0.32	1.82	0.80 - 4.13	0.18	0.07 - 0.48
Bengali	1.75	1.53 - 2.01	0.76	0.67 - 0.87	1.65	1.41-1.94	0.89	0.76 - 1.03
**Occupation**								
Agriculture	1		1		1		1	
Day labor	0.79	0.67 - 0.92	1.09	0.94 - 1.26	0.76	0.63 - 0.91	1.07	0.90 - 1.26
Service	1.32	1.08 - 1. 63	0.77	0.64 - 0.93	1.11	0.85 - 1.44	0.90	0.71 - 1.14
Business	1.23	0.84 - 1.79	1.15	0.75 - 1.47	1.01	0.67 - 1.51	1.14	0.79 - 1.63
Others	0.76	0.65 - 0.88	1.16	1.00 - 1.34	0.71	0.59 - 0.84	1.16	0.99 - 1.37

**Table 3:** Spatial cluster of treatment seeking approach in Rajasthali

Cluster	Household	No. of household	Expected household	Relative Risk	Log Likelihood Ratio	P-Value
**Most likely cluster for control programme**						
	2658	2453	1771	2.24	861.56	0.001
**Secondary clusters**						
	18	18	12	1.5	7.3	0.816
**Most likely cluster for Drug vendors**						
	1962	1649	957	2.97	827.25	0.001
**Secondary clusters**						
	86	78	42	1.9	35.22	0.001
	73	62	36	1.8	21.19	0.001
	62	54	30	1.8	20.49	0.001
	17	17	8	2.05	12.23	0.02
	16	16	7	2.05	11.51	0.04
	38	30	19	1.6	7.38	0.78
	9	9	4	2	6.46	0.99
	14	13	7	1.9	6.41	0.99

## Discussion

This is the first independent evaluation of a GFATM supported malaria control programme in a malaria endemic remote area of Bangladesh. According to published literature another study in Africa has examined GFATM supported malaria control programmes [21]. The analysis indicates significant levels of success in the delivery of interventions to the communities most at risk. However, a number of challenges still remain for the control of malaria in this region of Bangladesh.

**Figure 3:**
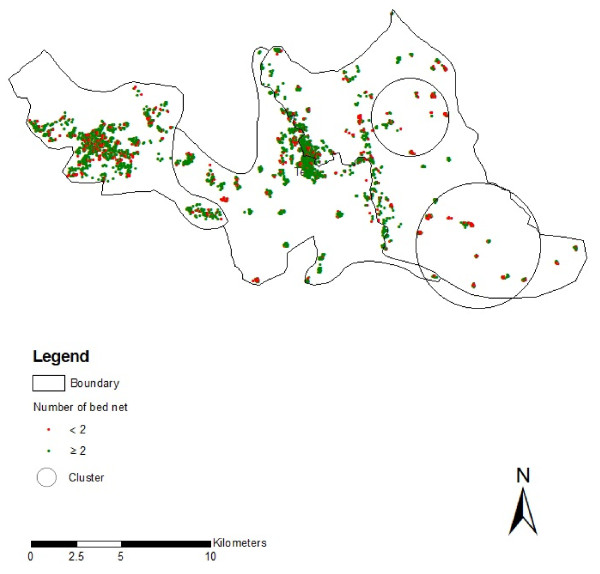
**Distribution of household clusters who have less than two bed nets**.

The target goal for the supply of LLIN was 80% of households in the malaria endemic areas [2] were surpassed as we have found that LLIN were supplied to 90.09% of households by BRAC. Additionally, during the study BRAC had treated 40.98% of household ITN/bed nets within the previous six months - substantially ahead of the two year timeline that was anticipated [2]. The final programmatic metric, based on the national control programme, was that every household should have more than two bed nets [2]. This goal has not yet been reached, with slightly more than a third of households lacking a second LLIN. These households tended to occur in more isolated regions. In hard to reach areas, a visualization approach using geographic information system (GIS) can be applied for target intervention. There were six significant clusters of households who have less than two bed nets (Table 4). Large clusters of households who have less than two bed nets were in the eastern part of the study area (Figure 3) where access is most limited by the landscape.

**Table 4:** Spatial cluster of insufficient bednets (< 2) in Rajasthali

Cluster	Household	No. of household	Expected household	Relative Risk	Log Likelihood Ratio	P-Value
**Most likely cluster**						
	344	166	81	2.15	49.79	0.001
**Secondary clusters**						
	102	60	24	2.56	29.48	0.001
	16	16	3	4.27	23.16	0.001
	18	15	4	3.55	14.40	0.006
	9	9	2	4.25	13.01	0.015
	19	14	4	3.14	10.64	0.082
	9	8	2	3.78	8.69	0.367
	6	6	1	4.24	8.66	0.545
	15	11	3	3.12	8.28	0.609
	38	20	9	2.24	7.48	0.711
	8	7	2	3.71	7.37	0.787
	8	7	2	3.71	7.37	0.787
	8	7	2	3.71	7.37	0.787
	5	5	1	4.24	7.22	0.959
	5	5	1	4.24	7.22	0.959
	5	5	1	4.24	7.22	0.959

In sub-Saharan Africa, recent studies show that 66% people prefer to be treated for malaria in a health care facility, 19% people prefer to buy drugs from a shop and a non-significant proportion of people take medicine from traditional healers, use herbal medicines, self-treatment or have no treatment [22]. Despite the overwhelming preference for health care facilities, and free treatment in government hospitals, people continue to use private clinic/drug vendors at high rates [23]. Results obtained in south-eastern part of Bangladesh are similar to that of sub-Saharan Africa. The widespread distribution of BRAC health workers provide better coverage than other health service providers and they can provide treatment to the majority of the population. However, at a relatively short distance (2 km) individuals appear to choose alternative health care sources. Nearly half of the people preferred drug vendors who provide anti-malarial drugs for fever without diagnosis. In the 2007 malaria baseline survey in the CHT districts, it was found only 51.5% (103/200) fever cases were positive for malaria. Total subjects enrolled in CHT for that survey was 2250. This suggests a significant potential for improper treatment with ACT or other anti-malarials from drug vendors in the region.

It is still unclear why people continue to prefer to pay drug vendors for treatment when the malaria control programme provides diagnosis and treatment in the community at free of cost.

## Conclusion

The target goal for the supply of LLIN and retreatment of ITN were surpassed. Distance from the national malarial control programme facilities may be a factor although some people preferred to receive treatment from a drug vendor rather than the malaria control programme, even if the drug vendor was more than 2 km away. This suggests that factors related to social interactions with the vendor, such as history, consistency of availability or other factors may come into play. At least some of these factors may be associated with ethnic experiences and the bases for these clustering's need further examination. Unsurprisingly educational experiences were associated with preferences for the use of malaria control programme services. A high proportion of people preferred drug vendors without having a proper diagnosis. Drug vendors are highly patronized and thus there may be the need to improve their services for public health good. Otherwise it may cause incomplete treatment, misuse of anti-malarial drugs, contribute to the risk of drug resistance and jeopardize the present malaria control efforts in Bangladesh.

## Conflict of interests statement

The authors hereby certify that no conflict of interest of any kind occurred in the framework of this study.

## Authors' contributions

UH was responsible for the design of the study, data preparation, analysis and interpretation of the data, and produced the final report. MH and TS was responsible for data preparation, analysis and final interpretation. GG, RH, SIH, SMA, SH, TY was responsible for the conception, overall scientific management, interpretation of data, and preparation of the final report. All authors read and approved the final manuscript.

## Supplementary Material

Additional file 1 **Supplemental appendix**. Data collection method. Click here for file
