# Supplementary material for: Progress and challenges to control malaria in a remote area of Chittagong hill tracts, Bangladesh
Source: Malar J. 2010 Jun 10;9:156. doi: 10.1186/1475-2875-9-156 (PMC2910016; doi:10.1186/1475-2875-9-156)
Supplement: Additional file 1 — Supplemental appendix. Data collection method. [file 1475-2875-9-156-S1.DOC]

**APPENDIX**

**Data collection method**

Four trained, educated and experienced young tribal males were recruited to collect information from households. They had considerable experience of performing malaria prevalence surveys and could speak in Bangla and other tribal languages. Since different tribes live in the study area, one person from every community was recruited to help implement the census. A 1-week training session was conducted to clarify everything to the interviewers. Training was also given on how to operate GPS and collect latitude and longitude data to locate households, health workers’ position, and health facilities in and outside Rajasthali.

The survey was started on 7^th^ January, 2009 and finished at the end of April 2009. Many areas were accessible only by foot because the tribes live on hills in clusters, and in some cases, it took two days to reach the area being censused. A working schedule was prepared one week in advance in the field office. A team of two people went on 5-days mission. Before departure, they took sufficient questionnaires, batteries, GPS, pens, pencils and other necessary items. As soon as they reached a community, they met with some village people or the tribal leader to discover how many families lived in that village. Upon completing the survey work, they returned to the tribal leader to check details of missing households. After completing the work, they spent the night in the local tribal leader’s house. After completing 5 days mission, they returned to the field office. Their supervisor performed an exhaustive check of the completed survey forms and entered information in the computer database system. All incomplete and suspected forms were separated and sorted according to village. As soon as the whole survey was complete, the workers returned to villages to resolve questions.
